# Use of Alfentanil in Palliative Care

**DOI:** 10.3390/pharmacy8040240

**Published:** 2020-12-16

**Authors:** José António Ferraz Gonçalves, Filipa Sousa, Lucy Alves, Patrícia Liu, Sara Coelho

**Affiliations:** 1 Serviço de Cuidados Paliativos, Instituto Português de Oncologia, 4200-072 Porto, Portugal; 2 Serviço de Medicina Interna, Centro Hospitalar Tondela Viseu, 3504-509 Viseu, Portugal; filipasousa04@gmail.com; 3 Serviço de Oncologia Médica, Centro Hospitalar Universitário do Algarve, 8000-386 Faro, Portugal; lucyalves15@gmail.com; 4 Serviço de Oncologia Médica, Centro Hospitalar de Trás-os-Montes e Alto Douro, 5000-508 Vila Real, Portugal; patriciacliu@gmail.com; 5 Serviço de Oncologia Médica, Instituto Português de Oncologia, 4200-072 Porto, Portugal; saramarinacoelho@gmail.com

**Keywords:** alfentanil, pain relief, renal impairment, palliative care

## Abstract

Alfentanil is used for chronic pain relief in palliative care. However, there is a dearth of data on its use. For this reason, a decision was made to review the use of alfentanil in palliative care. Retrospective study was carried out in a palliative care service. The files of patients who received alfentanil as an intravenous or subcutaneous continuous infusion for pain relief, between January 2018 and April 2019. In total, 111 patients received alfentanil out of 113 admissions. Of them, 56 were male, and the median age was 70 years. The median number of days on alfentanil was 6 (range 1 to 129). The most frequent primary reasons for switching to alfentanil was uncontrolled pain in 52 (46%) patients and renal impairment in 24 (21%) patients. The median 24-h initial dose of alfentanil was 4 mg (1–20), and the median final 24-h dose of alfentanil was 5 mg (1–60), (*p* < 0.001). The initial 24-h median number of rescue doses was 2 (0–8), and the final median number of rescue doses was 1 (0 to 8), (*p* = 0.025). In 56 patients who were on alfentanil for at least 7 days, the dose decreased in 3 (5%), remained stable in 10 (18%) and increased in 43 (77%). The patient on alfentanil for 129 days maintained the same dose throughout that period. Alfentanil can be a useful second-line opioid. The induction of tolerance does not seem to be particularly rapid with alfentanil.

## 1. Introduction

In 1986, the World Health Organization (WHO) published “Cancer Pain Relief” where the principles of the pharmacological treatment of pain in patients with cancer were presented [1]. Opioids, mainly morphine, were considered very important drugs in that setting. However, the pharmacology of opioids differs depending on several factors, such as how they are metabolized and eliminated, individual metabolic rates, liver and kidney impairment and other comorbidities. Therefore, although morphine is still the most used opioid, in some situations, including when it is ineffective or causes unbearable toxicity, it must be switched (also referred as opioid rotation) to another opioid [2].

Alfentanil is a short-acting synthetic opioid chemically similar to fentanyl. It is an agonist of μ receptors usually used in anesthesia for surgery [3], for analgesia in painful procedures [4] and in some cases in palliative care for control of moderate to severe pain, mainly in patients with renal impairment [5].

Via the intravenous route, the time to onset of analgesia of alfentanil is 55.7 s (range 15 to 120) and the maximal analgesic effect occurs within 1 to 2 min [6]. It is metabolized mainly by the liver, primarily through the cytochrome P450 3A4 enzyme by *N*- and *O*-dealkylation and 70–80% of the metabolites are eliminated in the urine, with only 1% excreted unaltered via that route [6]. Drugs that inhibit Cytochrome P450 3A4, such as fluconazole or erythromycin, may also inhibit the metabolism of alfentanil and, in so doing, may increase the risk of toxicity.

The serum creatinine level is not an accurate method for assessing renal function. A study showed that 10% of cancer patients have an elevated serum creatinine level, but 60% have a creatinine clearance of less than 90 mL/min, estimated by the Cockcroft–Gault and the abbreviated Modification of Diet in Renal Disease (aMDRD) formulas [7]. Retrospective reports vouch for the safety of alfentanil use in patients with renal impairment [5,8]. However, renal function impairment was not the only reason for switching to alfentanil; in some cases, the reason was toxicity of the previous opioid or insufficient analgesia in patients unable to take oral medication [5].

There are contradictory data on the rapid induction of tolerance by alfentanil infusion [9], although the quality of the data is questionable.

Alfentanil has been used in our service for many years in patients with documented or suspected renal impairment and as an alternative when other opioids are ineffective or cause significant toxicity. The dose we use for conversion from subcutaneous morphine is 10:1. The recommendations for the conversion of diamorphine to alfentanil is 10:1 [8] in most cases, which makes the conversion of morphine to alfentanil equivalent to 15:1. However, a study suggests that the conversion ratio from diamorphine may be approximately 6:1 [10], which is equivalent to a ratio to morphine of approximately 10:1.

We decided to review our practice on the use of alfentanil as there is a dearth of reports on alfentanil for chronic pain relief, particularly in palliative care. This study may provide some more information to palliative care professionals.

## 2. Methods

We carried out a retrospective study in a 40-bed palliative care service at an oncology center. The files of the patients who received alfentanil as an intravenous or subcutaneous continuous infusion for pain relief, between January 2018 and April 2019, identified though the electronic system of the hospital, were reviewed. Patients who received alfentanil only as a rescue medication or for painful procedures were excluded.

In addition to the demographic variables, the data collected were on the reason for the use of alfentanil, the opioids used before alfentanil, the initial and final 24-h dose, the number of rescue doses used in the first 24 h and in the last 24 h of the treatment, drugs which interfere with CYP 3A4, any switch from alfentanil to another opioid and why, and the outcomes (discharge or death).

Descriptive statistical methods were used for analyzing the data. For the comparison between initial and final doses of alfentanil and the number of rescue doses, the Wilcoxon test was used. The level of significance was deemed to be 0.05.

This study was approved by the ethics committee of the hospital.

## 3. Results

During the period indicated, 111 patients received alfentanil out of 113 admissions. The gender distribution was almost 50/50 (55 females, 56 males). The median age was 70 years (range 36 to 90). The most frequent primary cancer was colorectal—23 (21%)—followed by gynecological cancer—12 (11%) (Table 1). The most frequent metastatic sites were lymph nodes—45 (41%)—followed by liver and lung metastases—38 (34%) each (Table 1).

The median number of days on alfentanil was 6 (range 1 to 129). The opioids most often used before alfentanil were morphine—53 (47%)—and transdermal fentanyl—15 (13%) (Table 2). The most frequent primary reason for switching to alfentanil was uncontrolled pain in 52 (46%) patients, followed by renal impairment in 24 (21%) patients (Table 3); in 22 cases, there was more than one reason. The 24 patients with documented renal impairment had a median creatinine clearance, calculated using the aMDRD formula, of 21.45 mL/min (range 5.90 to 45.00). In 92 cases, other drugs were also used for pain relief, with corticosteroids being the main pharmacologic group, followed by anticonvulsants (Table 4). The rescue opioids were morphine in 75 (66%) cases, alfentanil in 23 (20%) and fentanyl in 4 (4%) and the routes used were subcutaneous in 91 (81%), oral in 5 (4%), sublingual in 4 (4%) and intravenous in 2 (2%). The median 24-h initial dose of alfentanil was 4 mg (1 to 20), 75 (66%) subcutaneously and 38 (34%) intravenously, and the initial 24-h median number of rescue doses was 2 (0 to 8). The median final 24-h dose of alfentanil was 5 mg (1 to 60), which was significantly higher than the median initial dose (*p* < 0.001), and the median number of rescue doses was 1 (0 to 8), which was significantly lower than the initial number (*p* = 0.025). There were only five cases where a drug influencing the cytochrome P450 3A4 isoenzyme was not used simultaneously, and the most frequent ones were midazolam and a corticosteroid; in many cases more than one was used.

**Figure 1:**
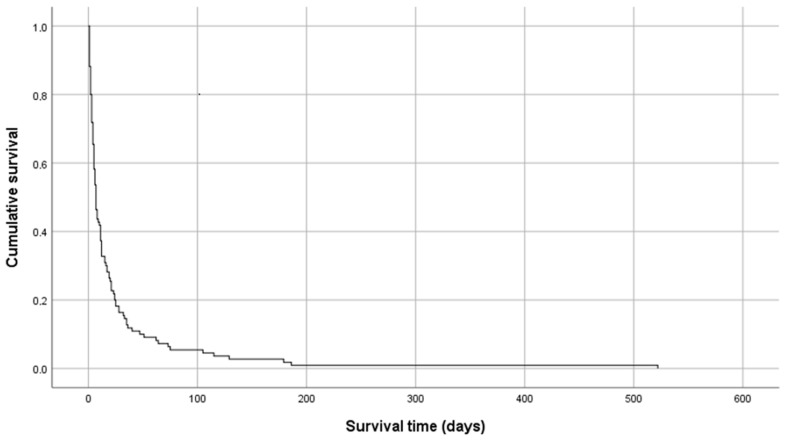
Overall survival.

In 21 cases, alfentanil was switched to other opioid: 10 to transdermal fentanyl, 5 to transdermal buprenorphine, 2 to oral morphine, 2 to subcutaneous sufentanil, 1 to oral hydromorphone and 1 to oral methadone. The most frequent reason for switching to another opioid was discharge in 15 (71%) cases, inefficacy in 2 (10%) and not stated in 4 (19%). In 15 (13%) cases, patients were discharged and 98 (87%) died in the palliative care service. Only one patient was discharged on a continuous subcutaneous infusion of alfentanil. The median overall survival was 7 days, with a range of 1 to 521 days (Figure 1).

We also analyzed the group of patients who were on alfentanil for at least seven days. Fifty-six patients were on alfentanil for at least 7 days, the median initial daily dose was 4 mg (1 to 20) and the median final daily dose was 10 mg (1 to 60) (*p* < 0.001). The median number of rescue doses was: initial—2 (0 to 7); final—1 (0 to 5) (*p* = 0.007). In 13 (23%) patients, alfentanil was switched to another opioid: 8 to transdermal fentanyl, 2 to sufentanil, and 1 each to transdermal buprenorphine, oral hydromorphone or methadone. The reasons for switching were preparation for discharge in 9 cases, inefficacy in 1 and not stated in 3. Eight (14%) patients were discharged and 48 (86%) died in the palliative care service. Of these 56 patients, the dose decreased in 3 (5%), remained stable in 10 (18%) and increased in 43 (77%). The patient who took alfentanil for 129 days maintained the same dose throughout that period. On the other hand, a patient on alfentanil for 35 days had the dose increased from 3 mg/day to 60 mg/day (2000%).

## 4. Discussion

Alfentanil is used in palliative care, usually as a second-line opioid. As a rapid onset and short-acting opioid, it is more suitable for continuous infusion, either subcutaneous or intravenous, for rescue doses for breakthrough pain or for painful procedures [9].

We analyzed all patients treated with alfentanil over a 16-month period and, specifically, we also analyzed the 56 patients who were treated for at least one week, as a longer period would be more informative.

Renal function impairment is the reason most often indicated for the use of alfentanil in advanced cancer [5,8,9], as the metabolites of alfentanil are deemed to be inactive, although the evidence for this is very limited [9], and only a small percentage is excreted unchanged in urine. It should be noted that when switching from opioids influenced by renal function to others not influenced by this, or vice versa, the usual conversion factors should not be directly used. However, there are other reasons for switching the previous opioid to alfentanil, such as uncontrolled pain or toxicity, as was the case in this study and also in another one carried out in the palliative care setting [5]. In fact, alfentanil may be an alternative opioid to switch to when the previous opioid is ineffective or induces severe undesired effects.

Alfentanil is metabolized by the cytochrome P450 (CYP) 3A4 enzyme, which is the most prevalent CYP enzyme in the human liver and is involved in the metabolism of over 50% of drugs [11]. Therefore, like all drugs metabolized via this route, its levels may be influenced by many other drugs, which may accelerate or retard its metabolism. This means that the effect of alfentanil should be carefully monitored in terms of analgesia and toxicity. In this study, it was not possible to observe such an influence, as it was also not possible in the other study [5]. However, this requires prudence which should not be applied solely to alfentanil, as most drugs are metabolized by this enzyme.

Another aspect is the possible occurrence of faster development of tolerance with alfentanil than with other opioids [12]. Data from this study seem to contradict that possibility, as 18% and 5% of patients on alfentanil for at least one week maintained the same dose or decreased the dose, respectively. The patient who was on alfentanil for the longest period maintained the same dose for 129 days. On the other hand, one patient on a 35-day period had the dose increased by 2000%. In 77%, the dose was increased, but this may imply only the normal titration of alfentanil. Therefore, the conclusion should be that the most important reason for changing the dose of alfentanil is the patient’s clinical situation rather than tolerance.

One problem with alfentanil concerns patients whose situation has stabilized and whose discharge is planned, as alfentanil must be given via the subcutaneous or intravenous route, which are not the most convenient routes for home administration. Therefore, in most patients in this situation, a switch to another opioid administered via oral or transdermal routes should be made. Drugs should be selected carefully and tested before discharge, mainly in patients with renal impairment. However, it is not impossible to administer alfentanil at home via continuous infusion, provided the proper assistance is available, as was the case with one patient.

As this is a retrospective study, it is not possible to consistently capture all the aspects which would be relevant, mainly the effectiveness of alfentanil in pain relief and the toxicity produced. However, the significantly lower number of rescue doses of analgesics during the period of utilization of alfentanil suggests that it is an effective opioid for pain relief in palliative care.

## 5. Conclusions

This study shows that alfentanil can be a useful second-line opioid and, in patients with advanced renal impairment, may be particularly useful. In these patients it may even be considered as a first-line opioid. However, as it must be administered as a continuous infusion it is not the most useful opioid for home care, although a subcutaneous continuous infusion may be possible at home, in selected cases, with the support of a home care team. Rapid induction of tolerance does not seem to be a particular problem with alfentanil as, in some cases, the same dose can be maintained for a relatively long period.

## Figures and Tables

**Table 1:** Demographic data.

Primary Cancer	*n*	%
Colorectal	23	21
Gynecological	12	11
Stomach	10	9
Prostate	8	7
Lung	8	7
Head and neck	6	5
Breast	5	5
Other	39	35
Total	111	100
**Metastases**		
Lymph nodes	45	41
Liver	38	34
Lung	38	34
Bone	35	32
Locally advanced	33	30
Peritoneum	21	19
Brain	8	7
Other	23	21

**Table 2:** Opioids used before alfentanil.

	*n*	%
Morphine	53	47
Fentanyl	15	13
Buprenorphine	14	12
Tramadol	12	11
Hydromorphone	6	5
Methadone	2	2
Tapentadol	2	2
None	9	8
Total	113	100

**Table 3:** Reasons for switching to alfentanil.

	*n*	%
Uncontrolled pain	52	46
Renal failure	24	21
Toxicity	15	13
Other	18	16
Unknown	4	4

**Table 4:** Other drugs used for pain control.

	*n*	%
Corticosteroid	84	74
Anticonvulsant	17	15
Antidepressant	12	11
NSAID	4	4
Paracetamol	4	4
Other	3	3
